# Supplementary material for: Breathing dissipative solitons in optical microresonators
Source: Nat Commun. 2017 Sep 29;8:736. doi: 10.1038/s41467-017-00719-w (PMC5622060; doi:10.1038/s41467-017-00719-w)
Supplement: Supplementary file 5 — Description of Supplementary Files [file 41467_2017_719_MOESM5_ESM.pdf]

Description of Supplementary Files

File Name: Supplementary Movie 1
Description: Animated spatiotemporal evolution of two irregularly breathing solitons. This video showing the complete spatiotemporal evolution of the trace displayed in Figure 5g of the main manuscript. The horizontal axis spans over 3 breathing periods and and is moved by one breathing period from one movie frame to the next. Here, a breathing two-soliton state undergoes a transition to a breathing single-soliton state (switching). In spite of unstable breathing conditions, the relative breathing phase between the two solitons is maintained close to quadrature.

File Name: Supplementary Movie 2
Description: Animated spatiotemporal evolution of two irregularly breathing solitons. This video showing the spatiotemporal evolution of a breathing two-soliton state undergoing a transition to a breathing single-soliton state (switching). The horizontal axis spans over 3 breathing periods and and is moved by one breathing period from one movie frame to the next. In spite of unstable breathing conditions, the relative breathing phase between the two solitons seems to be maintained close to in-phase.

File Name: Supplementary Movie 3
Description: Animated spatiotemporal evolution of two irregularly breathing solitons. This video showing the spatiotemporal evolution of a breathing two-soliton state at the breathing onset. The horizontal axis spans over 3 breathing periods and and is moved by one breathing period from one movie frame to the next. The breathing synchronisation is not complete and the phase continuously accumulates, but the breathing tends to synchronise when close to in phase.

File Name: Peer Review File
